# Characterization and Coexpression Analysis of the *TIFY* Family Genes in *Euryale ferox* Related to Leaf Development

**DOI:** 10.3390/plants12122323

**Published:** 2023-06-15

**Authors:** Lanruoyan Xu, Ailian Liu, Tianyu Wang, Yuhao Wang, Liangjun Li, Peng Wu

**Affiliations:** College of Horticulture and Landscape Architecture, Yangzhou University, Yangzhou 225000, China; xulanruoyan@163.com (L.X.); liuailianyz@163.com (A.L.); 18852712804@163.com (T.W.); wangyuhaoyzu@163.com (Y.W.); ljli@yzu.edu.cn (L.L.)

**Keywords:** conserved synteny, evolutionary pattern, gene expression pattern analysis, *E. ferox*

## Abstract

TIFYs are plant-specific transcription factors that contain the TIFY structural domain and play an important role in plant leaf growth and development. However, the role played by TIFY in *E. ferox* (*Euryale ferox* Salisb.) leaf development has not been investigated. In this study, 23 *TIFY* genes were identified in *E. ferox*. Phylogenetic analyses of the *TIFY* genes showed clustering into three groups (JAZ, ZIM, and PPD). The TIFY domain was shown to be conserved. JAZ was mainly expanded via wholegenome triplication (WGT) in *E. ferox.* Based on analyses of the *TIFY* genes in nine species, we found that JAZ has a closer relationship with PPD, in addition to appearing the most recently and expanding most rapidly, leading to the rapid expansion of TIFYs in *Nymphaeaceae*. In addition, their different evolution types were discovered. Different gene expressions showed the distinct and corresponsive expression patterns of the *EfTIFYs* in different stages of tissue and leaf development. Finally, The qPCR analysis revealed that the expression of *EfTIFY7.2* and *EfTIFY10.1* showed an upward trend and high expression throughout leaf development. Further co-expression analysis indicated that *EfTIFY7.2* might be more important for the development of *E. ferox* leaves. This information will be valuable when exploring the molecular mechanisms of *EfTIFY*s in plants.

## 1. Introduction

TIFY family members play important roles in plant growth and development and stress responses [1,2]. In *Arabidopsis*, JAZ is involved in JA hormonal responses. The subfamily that contains JAZ also contains the Jas domain [3,4], which involves interactions with basic helix–loop–helix (bHLH) and myeloblastosis (MYB) transcription factors that regulate different JA-dependent responses [5,6,7,8,9]. The jasmonate-insensitive phenotype is a result of the overexpression of a truncated JAZ1 or the mutant of JAI3/JAZ3 gene (AT3G17860) in *Arabidopsis* [10]. The importance of CORONATINE-INSENSITIVE1 (COI1) in JA signaling, such as null mutations at this locus, is that it abolishes JA responses in diverse plant species [11]. In the presence of JA, JAZ proteins are targeted by the SCFCOI1 complex for ubiquitination and degradation, which consequently relieves the repression and rapid activation of JA responses [10,12,13]. AtTIFY10b/JAZ1 is degraded to stop the repression of JA responses by the SCFCOI1-dependent 26S proteasome pathway [14]. In addition, JAZ6 (AT1G72450) and JAZ9 (ATIG70700) have been shown to be redundant with JAZ1 in the JA signaling pathway [13,15]. AtTIFY9 is silenced by RNAi, which increases MeJA sensitivity [16]. Recently, regarding JAZ10, it was discovered that homo- and heteromeric interactions between most *Arabidopsis* JAZs are mediated by the conserved TIFY motif within the domain [17].

TIFY is a particular plant-specific class, which has a conserved TIFY domain to encode proteins (Zinc finger protein expressed in Inflorescence Meristem) [18]. The highly conserved TIFY domain contains a core motif, TIF [F/Y]XG, in the protein [18]. AT4G24470 was the first identified *TIFY* gene, expressed in reproductive tissue in Arabidopsis thaliana [19]. ZIM contains a C2C2-GATA zinc-finger (CX2CX20CX2C) [19]. The definition of the TIFY, ZIM, and C2C2-GATA gene families was unclear for a long time [20,21], until comprehensive system analyses of sequences were conducted to clarify this issue. Finally, the TIFY domain-containing members were identified and named TIFY. Based on phylogenetic and gene structure analyses, the TIFY proteins were divided into three groups, namely, ZML, PPD, and JAZ, in Arabidopsis [18,22]. While all TIFY proteins bear a TIFY domain, those in the ZML subfamily contain CCT (CONSTANS, CO-like, TOC1) and ZML (GATA-zinc finger domain) domains. In contrast, both the PEAPOD (PPD) and JASMONATE ZIM-DOMAIN (JAZ) subfamilies lack GATA and CCT domains [23]. In addition to this, the JAZ subfamily also contains the conserved sequence of the Jas motif, which bears the characteristic motif SLX2FX2KRX2RX5PY [24]. PPD proteins bear a unique N-terminal PPD domain, as well as a divergent Jas motif that lacks the conserved PY at its C-terminus.

Although this family has been reported in many species, only a few of genes have been functionally analyzed in Arabidopsis. The first discovered TIFY gene, ZIM/TIFY1 (AT4G24470), is overexpressed using CaMV 35S, resulting in petiole and hypocotyl elongation [21]. ZML1/TIFY2b (AT3G21175) and ZML2/TIFY2a (AT1G51600) are highly homologous with ZIM/TIFY1 (AT4G24470), identified as having similar domain compositions in terms of their encoded proteins and expression patterns compared to ZIM; however, their functions still remain unclear [22]. In Arabidopsis, the mutations of PPD1/TIFY4a and PPD2/TIFY4b show a leaf dome shape and an increased size. Overexpression of PPD could reduce the lamina size and promote silique development [24,25].

*Euryale ferox* is part of the angiosperm basal plant family *Nymphaeaceae* and has nutritional value for humans. It has also undergone triplication events dating back to ~17 million years ago (MYA). Recent completion of the *Euryale ferox* genome sequencing can now allow the genome-wide identification of the TIFY gene family [26]. The TIFY gene family has been extensively investigated in many sequenced plants [18,27]. However, further analyses of the evolutionary divergence, gene retention and fractionation, and genome-wide expression patterns of this gene family in *Euryale ferox* are needed.

In the present study, we identified 23 *EfTIFY* genes in *E. ferox* via a database search and analysis of the phylogenetic relationships, conserved motifs, and retention *TIFY* genes and *N. colorata TIFY* genes. We further studied the syntenic gene pairs, gene structure, duplication, and tissue-specific expressions of *EfTIFYs* in different tissues. Through transcriptome and qPCR analyses, key candidate TIFY genes closely related to leaf development were identified, which will provide a theoretical basis for analyzing the molecular mechanism of leaf development in *E. ferox*.

## 2. Results

### 2.1. Identification of TIFY Genes in E. ferox

**Figure 1:**
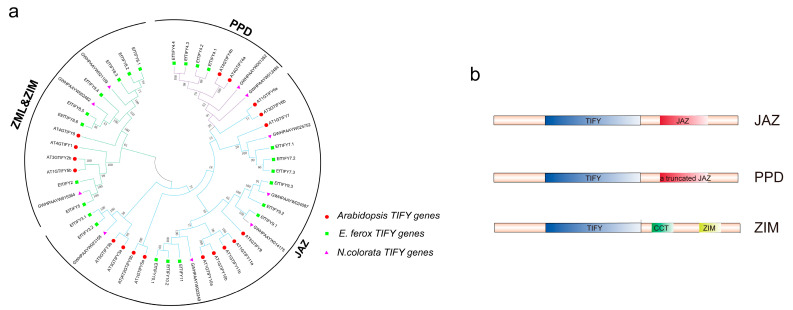
Phylogenetic analysis and protein domains in TIFY. (**a**) Phylogenetic tree of *E. ferox*, *Arabidopsis* and *N. colorata* TIFY; (**b**) Protein domains of three TIFY subfamilies. The red circle represents the TIFY in *A. thaliana*, the green square represents the TIFY in *E. ferox*, and the rose represents the TIFY in *N. colorata*.

A HMM for the TIFY domain identified 25 gene candidates in the *E. ferox* genome. Subsequently, by using Pfam and SMART, we found that two of the 25 putative *TIFY* genes lacked TIFY domains. Therefore, 23 genes in *E. ferox* were identified as TIFY family members (Supplementary Table S2). Three conserved domains were identified in the *EfTIFY* genes, including TIFY, Jas, and GATA. In particular, TIFY was more conserved, comprising approximately 40 amino acids. Jas and GATA were located in the C-terminus of the proteins. TIFYs can be divided into three subgroups according to their structural domains. JAZ contains TIFY and JAZ structural domains, PPD contains TIFY and truncated JAZ structural domains, and ZIM contains TIFY, CCT and ZIM structural domains (Figure 1b).

The chemical characteristics of EfTIFY by ExPASy were predicted (Supplementary Table S2); the theoretical pI of the JAZ subfamily was approximately nine, but five EfTIFY proteins (EfTIFY3.2, EfTIFY4.4, EfTIFY10.2, EfTIFY10.1, and EfTIFY3.1) showed a low pI of nine. The other classes had complex theoretical pI values, ranging from four to eight. EfTIFY proteins are unstable proteins, based on calculation of the average instability index being approximately 68.11. All EfTIFY proteins are hydrophilic, as their hydropathicity values were shown to be <0.

### 2.2. Phylogenetic and Classification Analysis of EfTIFY

**Figure 2:**
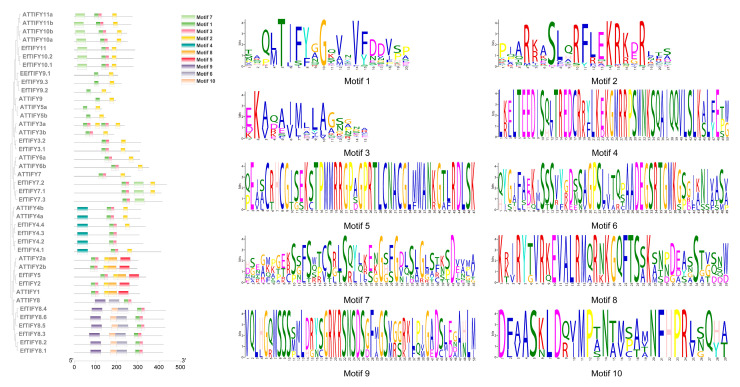
Phylogenetic relationships and conserved motif compositions of *E. ferox* and *Arabidopsis* TIFY proteins. The neighbour-joining tree of *E. ferox* and *Arabidopsis* TIFY genes and their motif locations. Introns and exons are represented by green dashed lines and colored boxes, respectively.

To investigate the classification and expansion of the *TIFY* genes in *E. ferox*, *N. colorata*, and *A. thaliana*, we conducted phylogenetic analyses of all of the *TIFY* genes. The phylogenetic analyses indicated that the EfTIFY family can be divided into three subfamilies (ZIM and ZML, PPD, and JAZ). JAZ has the largest number of *EfTIFY* genes (50%) (Figure 1a). To investigate the structural characteristics of the TIFY family in *E. ferox*, we chose TIFY of *A. thaliana* as a comparison. In the *E. ferox* and *Arabidopsis* TIFY proteins, we identified 10 conserved motifs (Figure 2). Genes from the same subfamily that share similar motif compositions are likely to share similar functions. Through a comparison of the *Arabidopsis* and *E. ferox* TIFY proteins, we found that they have similar structures in every subgroup. Moreover, each class of the EfTIFY proteins had several special motifs, such as motif 5, located at the N-terminal, which only exists in JAZV, and motif 4, located at the C-terminal, which only exists in PPD. All of the EfTIFY proteins contained motif 1, corresponding to the TIFY conserved domain. Motif2 corresponds to the CCT structural domain and motif5 corresponds to the GATA domain. These domains are the key to the function of *TIFY* family genes. The gene structure analysis indicated that most *EfTIFY* genes have at least three introns. As shown in Figure S1, the ZML and PPD subfamilies contained relatively more introns, meaning that they were stable and conserved during the evolution process [28].

### 2.3. Differential Copy Number and Retention of EfTIFY

To investigate the copy number variation in TIFY during *E. ferox*-specific WGT events, we compared the homologous *TIFY* genes in *N. colorata* and the three *E. ferox* sub-genomes (Sub1, Sub2, and Sub3) and found most EfTIFYs on the conserved collinear blocks (Supplementary Table S3). Specifically, compared to the PPD and JAZ genes, only the ZIM genes have been retained in three copies. The majority of the ZIM (100%) genes were retained in two or three copies, compared to only 83.33% of the JAZ genes and 50.00% of the PPD genes. It is known that the JAZ and ZIM proteins have many important functions that negatively regulate JA signaling via direct interaction with several transcription factors, according to the gene dosage hypothesis. In addition, the percentage of homeologs varies among the three sub-genomes. Compared to the other group of genes, significantly more *TIFY* gene homologs were retained in the Sub2 and Sub3 sub-genomes. AKE (ancestral karyotype of *E. ferox*) (AKE1-12) has been inferred in *E. ferox* [29,30]. Most of the 23 *EfTIFY* genes belong to AKE3 (43.4%), followed by AKE5 (21.7%), while only 4% of the *EfTIFY* genes are assigned to AKE11.

### 2.4. Expansion and Evolution of the TIFY Genes in Plants

**Figure 3:**
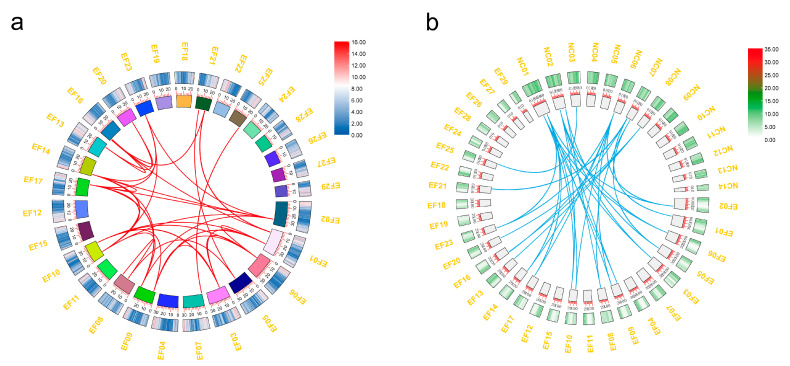
*EfTIFY* syntenic gene pairs between *E. ferox* and *N. colorata*. (**a**) The syntenic gene pairs *TIFY* genes in *E. ferox*; (**b**) The syntenic gene pairs *TIFY* genes between *E. ferox and N. colorata.* The scale on the figure is the length of the chromosome.

Comparative genomic analysis confirmed that *E. ferox* underwent genome triplication since its divergence from *N. colorata* [26]. For the analyses of the *EfTIFY* genes’ triplication in *N. colorata* from *E. ferox*, the syntenic gene pairs were analyzed. We identified 37 syntenic orthologous gene pairs using the MCScanX program among all of the TIFY proteins of *E. ferox*. Additionally, 33 syntenic orthologous *TIFY* gene pairs were identified between *E. ferox* and *N. colorata* (Figure 3). Among the syntenic orthologous gene pairs, we found that each *NcTIFY* gene had two or three *E. ferox* syntenic orthologous genes, demonstrating that the *TIFY* genes in *E. ferox* underwent duplication accompanied by genome triplication. However, the gene number in the *E. ferox* genome was notably three times lower than that of the *N. colorata*, indicating the gene loss that occurred during the polyploid process. The visualization of the syntenic orthologous genes among the two species was carried out using the TBtool software (Figure 3). Among the syntenic orthologous gene pairs between *E. ferox* and *N. colorata*, we found more *E. ferox TIFY* genes in *N. colorata* chromosomes 1 and 2. Furthermore, all of the *EfTIFY* genes were duplicated by the WGD or segmental events (Supplementary Table S4), through WGD or segmental duplication, to promote the expansion of the EFTIFY in *E. ferox*.

### 2.5. Expansion and Evolution Pattern of the TIFY Genes in Plants

**Figure 4:**
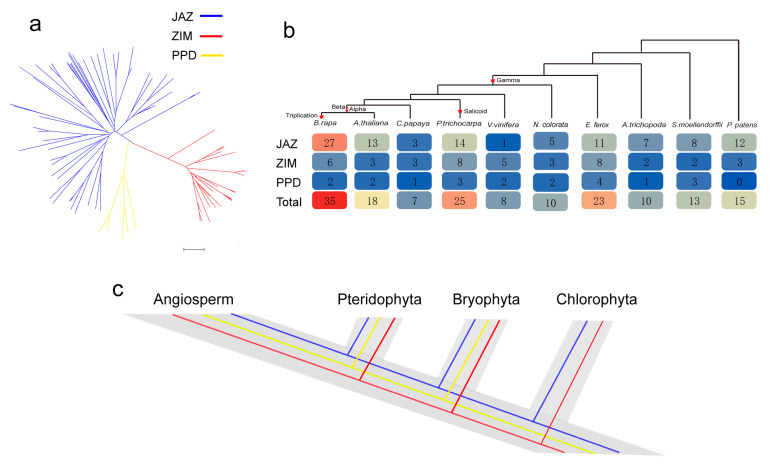
The dissection of *TIFY* genes evolution. (**a**) Phylogenetic tree of *TIFY* genes in ten plants; (**b**) Comparisons of the number of *TIFY* gene familys in ten plants. (**c**) The evolutionary pattern of TIFYs in plants.

To investigate the evolution of the TIFY family in the plant kingdom, we selected eight *Angiospermae* (seven eudicots and one basal angiosperm), one *Pteridophyta*, and one *Bryophyta* species for comparative analysis (Figure 4a). These plants have played an important role in evolution, such as polyploidization and duplication events [31,32,33]. The phylogenetic tree showed that TIFY also formed three distinct clades (JAZ, ZIM, and PPD). JAZ and ZIM were found to exist in *P. patens*, which indicates that these two groups originated from before *Bryophyta* diverged from *Chlorophyta*. However, the PPD subfamily was lost in the species of *Bryophyta*. Meanwhile, we found that no PPD was detected in *P. patens*. JAZ has a closer relationship with PPD, meaning that JAZ and PPD may share a common evolutionary origin, based on previous reports [18].

Furthermore, the number of *TIFY* genes in polyploidization plants was higher than that in the other species, and was most likely due to WGDs, which lead to gene family expansion. The *TIFY* gene family incurred WGT events in *E. ferox* and *B. rapa*, accounting for more than other species. Among the *TIFY* gene family, the JAZ subgroup has an important role in expansion, with the greatest rate and extent of expansion (Figure 4b). Compared to other groups, the number of PPDs is more stable. In summary, we infer a possible evolutionary footprint, with WGD being the main driving force in terms of the expansion and evolution of the *TIFY* gene family in plant genomes (Figure 4c).

### 2.6. Comparative Expression Pattern Analysis of the TIFY Genes between A. thaliana and E. ferox

**Figure 5:**
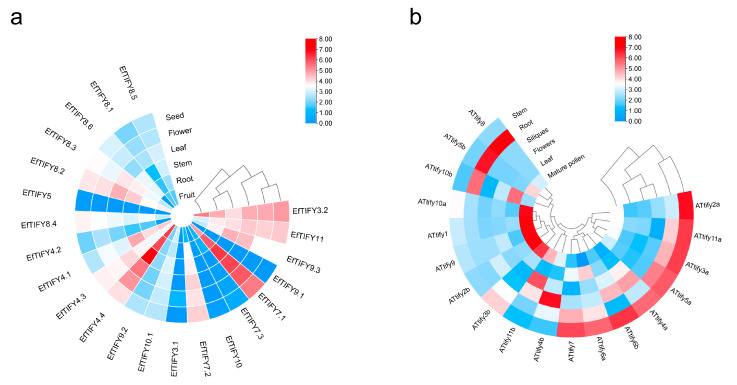
Expression analysis of *TIFY* genes. (**a**) Heatmap representation and hierarchical clustering of *EfTIFY* genes in root, stem, leaf, flower, seeds, and fruits; (**b**) Heatmap representation and hierarchical clustering of *AtTIFY* genes in root, stem, leaf, flower, siliques, and mature pollen.

The *TIFY* genes play important roles in plant growth involving physiological activity [18]. We investigated the different expression pattern of the *TIFY* genes in different tissues (roots, stems, leaves, and flowers) between *A. thaliana* and *E. ferox*. The seeds and fruits were studied only in *E. ferox*, and the siliques and mature pollen were studied only in *A. thaliana* (Figure 5 and Supplementary Table S7). The transcript levels (TPKM values) of 20 of the 23 *EfTIFYs* were obtained from at least one of the six tissues (Supplementary Table S6 and Figure 5). Interestingly, the TPKM values of five *EfTIFYs* (EfTIFY3.2, EfTIFY11, EfTIFY9.3, EfTIFY7.1, and EfTIFY7.2) in the JAZ subfamily were highly expressed, indicating that they may participate in the development of *E. ferox*. In addition, EfTIFY8.2 was highly expressed in the stems (Figure 5a). In *A. thaliana*, *AtTIFY8*, *AtTIFY85b*, and *AtTIFY10b* were highly expressed in the roots. Meanwhile, eight *TIFY* genes (*AtTIFY2a*, *AtTIFY11a*, *AtTIFY3a*, *AtTIFY4a*, *AtTIFY5a*, *AtTIFY6a*, *and AtTIFY6b*) were highly expressed in the stems. However, the homologs of *EFTIFY* were not highly expressed in the stems, such as EfTIFY7.3, EfTIFY10.2, and EfTIFY10.2. Furthermore, the paralogous genes were differently expressed due to the differentiation of function.

### 2.7. Differential Expression of the EfTIFY Genes under Leaf Development

**Figure 6:**
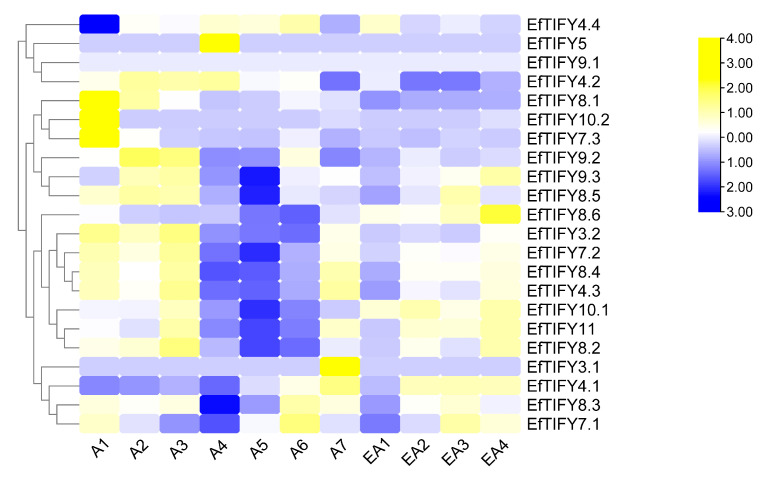
Expression analysis of *E. ferox TIFY* genes under different leaf development stages.

In the present study, we investigated the response of the *E. ferox* ZML, PPD, and JAZ subfamily genes to different leaf development processes (Figure 6, Supplementary Table S8). We further explored the gene pathways involved in the development of the early adult leaves (EA1–EA4) and adult leaves (A1–A7) development of *E. ferox*.

**Figure 7:**
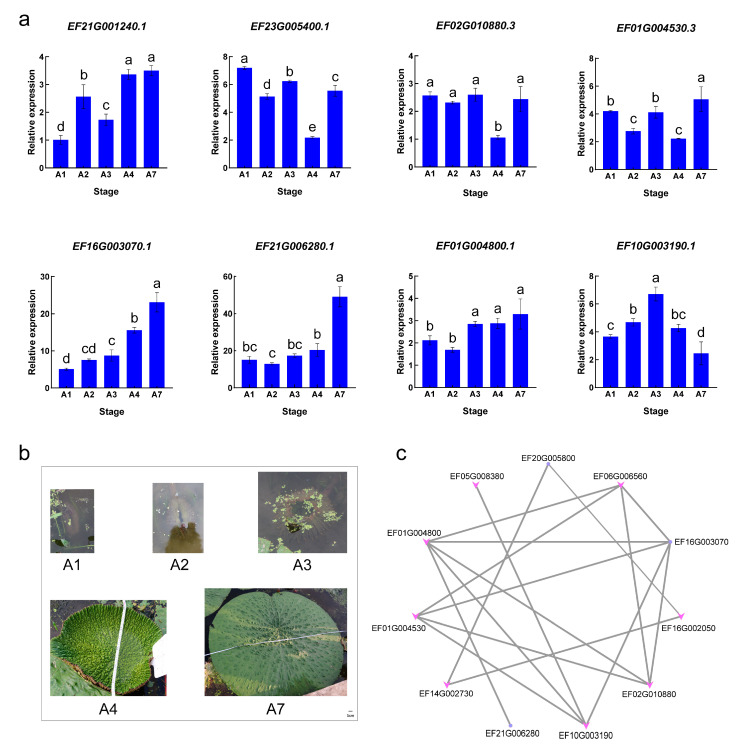
Expression analysis of *EfTIFYs* in *E. ferox* leaves during development and the construction of co-expression network. (**a**) qPCR analysis of EfTIFYs expression. (**b**) *E. ferox* leaves at different developmental stages (A1, A2, A3, A4 and A7 are respectively the diameters of the leaves of *E. ferox* are 3 cm, 15 cm, 25 cm, 50 cm and 200 cm). (**c**) Co-expression network of *EfTIFYs*. Data from three biological replicates were analyzed by ANOVA. Values with different letters are significantly different from each other (*p* < 0.05). Error bars show SD from three biological replicates.

All *EfTIFY* genes were lowly expressed at EA1 (Figure 6). Moreover, three *EFTIFY* genes (*EfTIFY4.1*, *EfTIFY10.1*, and *EfTIFY11*) were upregulated at EA2. As the leaves grow, more of the *EfTIFY* genes were highly expressed in EA3 and EA4. Specifically, we observed that the JAZ subgroup was highly expressed in the early adult leaves. For the adult leaves, we found that almost all of the *EfTIFY* genes had high expression levels at A1–A3, especially the JAZ subfamily, indicating a potentially important function in leaf development processes. In addition, *EfTIFY8.4*, *EfTIFY4.3*, *EfTIFY4.1*, and *EfTIFY10.2* were upregulated at A7. Furthermore, the expression pattern of *EfTIFYs* at critical stages of leaf development was analyzed via qPCR (Figure 7a,b). The expression of *EfTIFY9.2* was higher at the mature stage of leaf development (A4,A7), while the expression of *EfTIFY8.5* was high in submerged leaves (A1–A3), and the expression of *EfTIFY8.4* and *EfTIFY3.2* remained high during leaf development. The expression of *EfTIFY7.2*, *EfTIFY10.1* and *EfTIFY11* increased with leaf development, while the expression of *EfTIFY8.2* increased significantly in A1–A3 and decreased significantly in A3–A7 (Figure 7a, Supplementary Table S9). Notably, *EfTIFY7.2* and *EfTIFY10.1* had higher expression in A1 to A7 compared with other genes (Figure 7a, Supplementary Table S9), suggesting that these two genes may have critical roles in the growth and development of *E. ferox* leaves.

Plant cis-elements are important ways to regulate gene expression to participate in plant growth and development and adapt to the environment. Thus, we used the PlantCare online tool to identify TIFY genes of the cis-regulatory elements in Euryale ferox. Two cis-regulatory elements, ARBE and the CGTCA-motif, the GARE-motif, were responsive to plant hormones, including ABA and JA, in addition to cis-regulatory light-responsive element (AE-box and G-Box), low-temperature responsiveness (LTR), and drought-responsive element (MBS). These suggested that they could affect TIFY genes expression to regulate development in *Euryale ferox* (Figure S2).

In addition, a co-expression network was established for these *TIFY* genes with a Pearson correlation coefficient of exceed 0.8. *EfTIFY7.2* was observed to be significantly correlated with the expression of several genes (*EfTIFY11*, *EfTIFY3.2*, *EfTIFY8.2*, *EfTIFY8.4*), further confirming that this gene is likely to be a key gene in leaf development. Moreover, *EfTIFY11* was associated with several genes and its relative expression was high during leaf development. These results indicated that it is probable that multiple *TIFY* genes coordinated with each other to promote the growth and development of *E. ferox* leaves.

## 3. Discussion

*E. ferox* belongs to the basal *Nymphaeaceae* family, which is evolutionarily important for plants. It has recently been reported that the *TIFY* gene family plays an important role in plant development, which is tightly linked to stress responses [34]. A genome-wide identification method was performed to identify the *TIFY* gene in *E. ferox* and provide clues about its evolutionary history and expression diversification. Meanwhile, the expansion of *TIFY* in *E. ferox* and other plants was revealed. Through the expression profile data of different tissues and leaf development stages, it was revealed that the family genes have different tissue expression specificities and participate in *E. ferox* leaf development.

In this study, we identified 23 TIFY family genes in *E. ferox* and other species, including nine plants, one moss, one lycophyte, one mocnocotyledonous angiosperm, and six eudicotyledonous angiosperms. A total of 164 *TIFY* family genes were identified and analyzed in our study. The number of each subgroup in *E. ferox* is different to that in *N. colorata*, which is due to differences in replication and retention during evolution. Most of plant species have to undergo polyploidization [35,36]. Polyploidization is a major force in plant adaptive evolution [37,38]. Plants such as White lupin (*Lupinus albus*) have evolved from a whole-genome triplication (WGT) event. *Papaver* [39], *Acorus tatarinowii* [40] and *Saccharum spontaneum* [41] have also undegone polyploidization. As reported previously, *Euryale ferox* has also undergone triplication events during the last ~17 million years [26]. We found that angiosperms contain a relatively large number of *TIFY* genes. The *TIFY* genes exist in land plants, in which the TIFY domain originated after the divergence of algae from land plants and might have been essential in land plant emergence [18,22]. In this study, the number of JAZ subfamily genes in *E. ferox* was more than that in *N. colorata*, and the JAZ subfamily genes were preferentially retained relative to the other subfamily genes. Duplication events are the main factor driving gene family expansion in plants [19]. At the same time, gene retention and loss always occur in the evolutionary process, which varied in each TIFY subfamily gene.

The TIFY family is a novel, plant-specific gene family. In plants, the *TIFY* gene family plays very important roles in development, as well as in hormonal regulation and stress responses [1]. Gene expression patterns can provide important clues for gene function. An alternative splicing form of *JAZ10* (JAZ10.4) plays an essential role in the regulation of JA-induced degradation in *Arabidopsis thaliana* [42]. We speculate that *EfJAZ* transcription factors may also experience alternative splicing, and have an effect on the stability of the JAZ family and its function. Due to the close relationship between *E. ferox* and *Arabidopsis*, highly homologous genes between the two species were identified and used to predict the functions of the *TIFY* genes in *E. ferox*. For example, *AtTIFY1* (Supplementary Table S6) was reported to be involved in petiole and hypocotyl elongation. These results show that homologous *EfTIFY* genes may also function in the response to those abiotic and biotic stresses, which needs to be explored in further studies.

Furthermore, via qPCR analysis, we identified that the expression of *EfTIFY7.2* and *EfTIFY10.1* was closely related to the developmental process of the leaf, and these two genes are likely to play important roles in *E. ferox* leaf development.

## 4. Materials and Methods

### 4.1. Materials

The *Euryale ferox* plants were grown in Yangzhou University Aquatic Vegetable Test Base, normal cultivation management. Samples were taken at critical periods of leaf development (A1, A2, A3, A4, A7), snap-frozen in liquid nitrogen and stored at −80 °C in the refrigerator.

### 4.2. Identification of the TIFY Gene Family in Different Plants

The *E. ferox* genome was downloaded from NCBI [26], and we analyzed the domains using the HMM profile [43] of the TIFY domains (PF06200). The *Arabidopsis* TIFY sequences were used as the query to perform a BLAST search in these species, with a cutoff e-value of <10^−10^. To confirm the obtained proteins, the Pfam database (http://Pfam.sanger.ac.uk/, 1 January 2023) [44] and the SMART tool (http://smart.embl-heidelberg.de/, 1 January 2023) were used for the examination. All of the TIFY proteins were downloaded from the genome browser phytozome v13 (http://www.phytozome.net/, 1 January 2023) and were used for identification in the Pfam database.

### 4.3. Phylogenetic, Gene Feature, and Conserved Motif Analysis

MEGA5 was used to build phylogenetic trees via the neighbor-joining (NJ) method (bootstrap value of 1000) [17]. The MEME software was employed to identify motifs (http://meme.sdsc.edu/meme/, 1 January 2023) [45]. The gene structure was visualized using Gene Structure Display 2.0 (GSDS, http://gsds.cbi.pku.edu.cn/, 1 January 2023).

### 4.4. Identification of Syntenic Pairs of TIFY Genes in E. ferox

The Multiple Collinearity Scan toolkit (MCScanX) was used to identify syntenic gene pairs [46]. These syntenic gene pairs were then demonstrated with Circos using TBtool [47].

### 4.5. Gene Expression Data Analysis

To analyze TIFY in different tissues and different leaf development expression patterns in *E. ferox*, we used previously reported RNA-seq data [26]. Finally, heat maps of the hierarchical clustering were visualized using TBtool [47].

### 4.6. Quantitative Real-Time PCR Analysis

Plant RNA extraction kit (Takara, Dalian, China) was used to extract total RNA from leaf of *E. ferox* at different developmental stages. Then, HiScript^®^IIl RT SuperMixfor qPCR (Vazyme, Nanjing, China) was used to reverse transcription into cDNA. The qRT-PCR reaction was 20 μL, including 10 μL 2 × ChamQ SYBR qPCR Master Mix (Vazyme, Nanjing, China), 0.4 μL forward primer, 0.4 μL reverse primer, 1.0 μL cDNA template and 8.2 μL ddH_2_O, respectively. Primer Premier 5.0 was used for primer design. See Supplementary Table S1 for the gene specific primer sequences. *EfUBQ5-3* was used as an internal gene expression. Amplification was performed on the CFX-96 Real-time PCR system (Bio-Rad, Hercules, CA, USA) using the following real-time fluorescent quantitative PCR program: 95 °C for 30 s, then 95 °C for 10 s, and 60 °C for 30 s for a total of 40 cycles. The relative gene expression was calculated by 2^−ΔΔCT^ [48]. Three replicates were performed for each amplification reaction.

## 5. Conclusions

Bioinformatics plays a key role in examining the molecular regulation of *E. ferox* development, which provides us with basic resources, and the bioinformatics analysis results ensure the creditability of experimental results. In summary, 23 *TIFY* genes (seven ZIM genes, four PPD genes, and 12 JAZ genes) were identified in the entire *E. ferox* genome. A comparison of the phylogenetic relationships between *E. ferox* and *Arabidopsis TIFY* genes suggested that although most of the basic subfamilies have been retained in *E. ferox*, the number of each subfamily is different. We further demonstrated that WGD or segmental duplications have contributed to the expansion of the *TIFY* gene family. Comparative synteny analysis between the *E. ferox* and *N. colorata* genomes indicated that the majority of *E. ferox* and *N. colorata TIFY* genes are located in syntenic regions. Multiple *TIF*Y genes were identified via qPCR and co-expression analysis as possibly being involved in the leaf development process. These results will lay the foundation for resolving the molecular mechanism of leaf development in *E. ferox*.

## Data Availability

Data are contained within the article or in the Supplementary Materials.

## Supplementary Materials

The following supporting information can be downloaded at: https://www.mdpi.com/article/10.3390/plants12122323/s1.
