# Supplementary material for: Characterization and Coexpression Analysis of the TIFY Family Genes in Euryale ferox Related to Leaf Development
Source: Plants (Basel). 2023 Jun 15;12(12):2323. doi: 10.3390/plants12122323 (PMC10305134; doi:10.3390/plants12122323)
Supplement: Supplementary file 1 [file plants-12-02323-s001.zip › plants-2322244-supplementary.pdf]

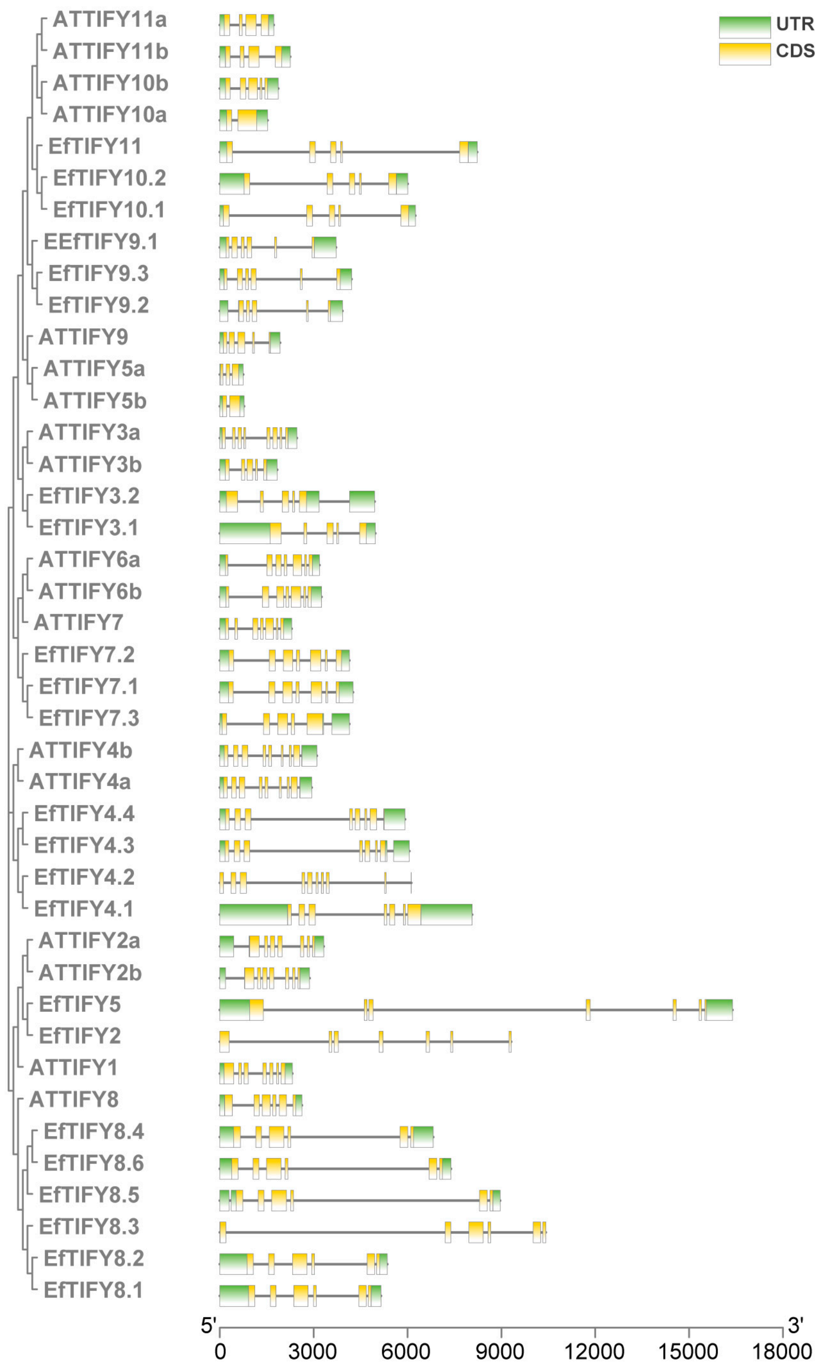

Figure S1. Structural analysis of *TIFY* gene in *E. ferox*.

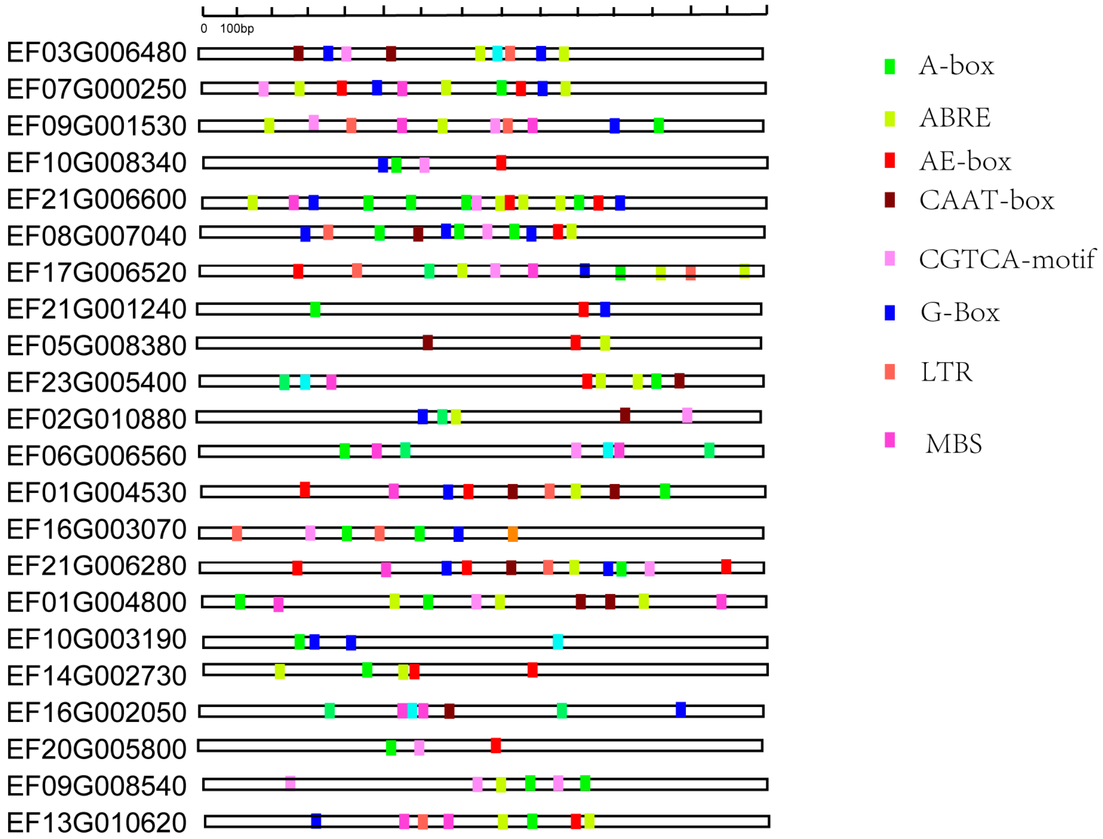

Figure S2. Analysis of the cis-element of the *TIFY* genes promoter.
